# Metastatic Urachal Carcinoma With Microsatellite Stability and Low Tumor Mutational Burden Achieving a Durable Response to Pembrolizumab After Platinum Failure

**DOI:** 10.1002/iju5.70276

**Published:** 2026-09-27

**Authors:** Fumihiro Ito, Koki Kobayashi, Gaku Hayashi, Shunsuke Kamijo, Takashi Fujita

**Affiliations:** ^1^ Department of Urology Gifu Prefectural Tajimi Hospital Tajimi Japan

**Keywords:** immune checkpoint inhibitor, pelvic lymph node metastasis, pembrolizumab, platinum chemotherapy, urachal carcinoma

## Abstract

**Introduction:**

Urachal carcinoma is a rare and aggressive malignancy with no established systemic therapy after platinum failure. Evidence for immune checkpoint inhibitors (ICIs) remains limited, particularly in tumors without established favorable biomarkers such as microsatellite instability‐high status or high tumor mutational burden.

**Case Presentation:**

A 55‐year‐old woman with metastatic urachal adenocarcinoma progressed after seven cycles of gemcitabine–cisplatin. Pembrolizumab (200 mg every three weeks) was initiated, resulting in a durable partial response lasting over 12 months without immune‐related adverse events. Molecular profiling showed microsatellite stability, low tumor mutational burden, and insufficient tissue for (Programmed death‐ligand 1) PD‐L1 assessment.

**Conclusion:**

This case demonstrates durable disease control with pembrolizumab in metastatic urachal carcinoma despite microsatellite stability and low tumor mutational burden. These findings suggest the potential efficacy of pembrolizumab in selected patients with platinum‐refractory disease.

## Key Clinical Message

Despite microsatellite stability and low tumor mutational burden, pembrolizumab achieved durable disease control in metastatic urachal carcinoma after platinum failure. This case suggests that PD‐1 blockade may have clinical activity even in the absence of MSI‐H or high TMB.

**Abbreviations**
CT: computed tomography
GC: Gemcitabine–cisplatin
ICI/ICIs: immune checkpoint inhibitor(s)
MSS: microsatellite stable/microsatellite stability
PD‐1: programmed cell death protein 1
PD‐L1: programmed death‐ligand 1
PD‐L2: programmed death‐ligand 2
TMB: tumor mutational burden
UrC: Urachal carcinoma

## 1 Introduction

Urachal carcinoma (UrC) is a rare malignancy accounting for less than 1% of bladder cancers [1]. It is often diagnosed at advanced stages [2]. No standardized systemic therapy exists for metastatic or platinum‐refractory disease [3, 4].

Gemcitabine–cisplatin (GC), extrapolated from urothelial carcinoma, has shown variable and generally short‐lived efficacy in UrC [5, 6, 7]. The role of radiotherapy remains unclear [8].

Immune checkpoint inhibitors (ICIs) such as pembrolizumab have demonstrated durable responses in bladder and other cancers [9], but data in urachal carcinoma are scarce, mostly in tumors with high PD‐L1 expression or elevated tumor mutational burden (TMB) [10, 11, 12]. The clinical value of ICIs in microsatellite‐stable, low‐TMB UrC remains uncertain [12].

This case demonstrates that pembrolizumab can provide durable benefit in metastatic urachal adenocarcinoma even in the absence of favorable biomarkers such as MSI‐H or high TMB.

## 2 Case Presentation

**FIGURE 1:**
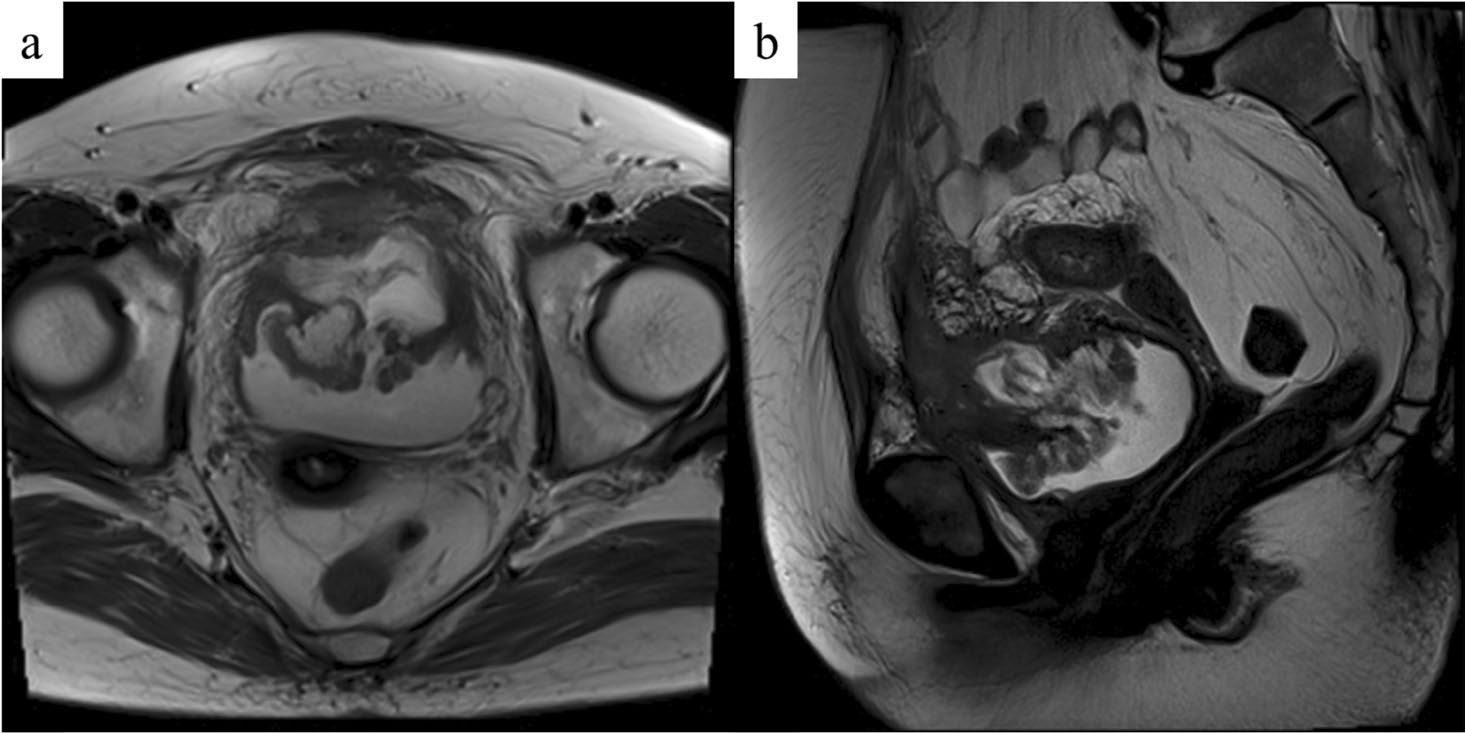
MRI findings at diagnosis. (a) Axial T2‐weighted image demonstrating a heterogeneous, predominantly hyperintense mass arising from the bladder dome, characterized by mixed solid and mucinous components. Nodular soft‐tissue lesions are present along the peritoneal surface, consistent with peritoneal dissemination. (b) Sagittal T2‐weighted image clearly showing a midline supravesical mass extending toward the urachal remnant with extravesical growth. Additional small nodules along the anterior peritoneum further support the presence of peritoneal implants.

A 55‐year‐old woman presented with dysuria and gross hematuria in September 2021. Cystoscopy revealed a bladder dome lesion, and transurethral resection demonstrated a mucin‐producing adenocarcinoma consistent with urachal origin. MRI showed a midline supravesical mass extending from the umbilicus to the bladder dome (Figure 1a,b), suggesting urachal carcinoma.

**FIGURE 2:**
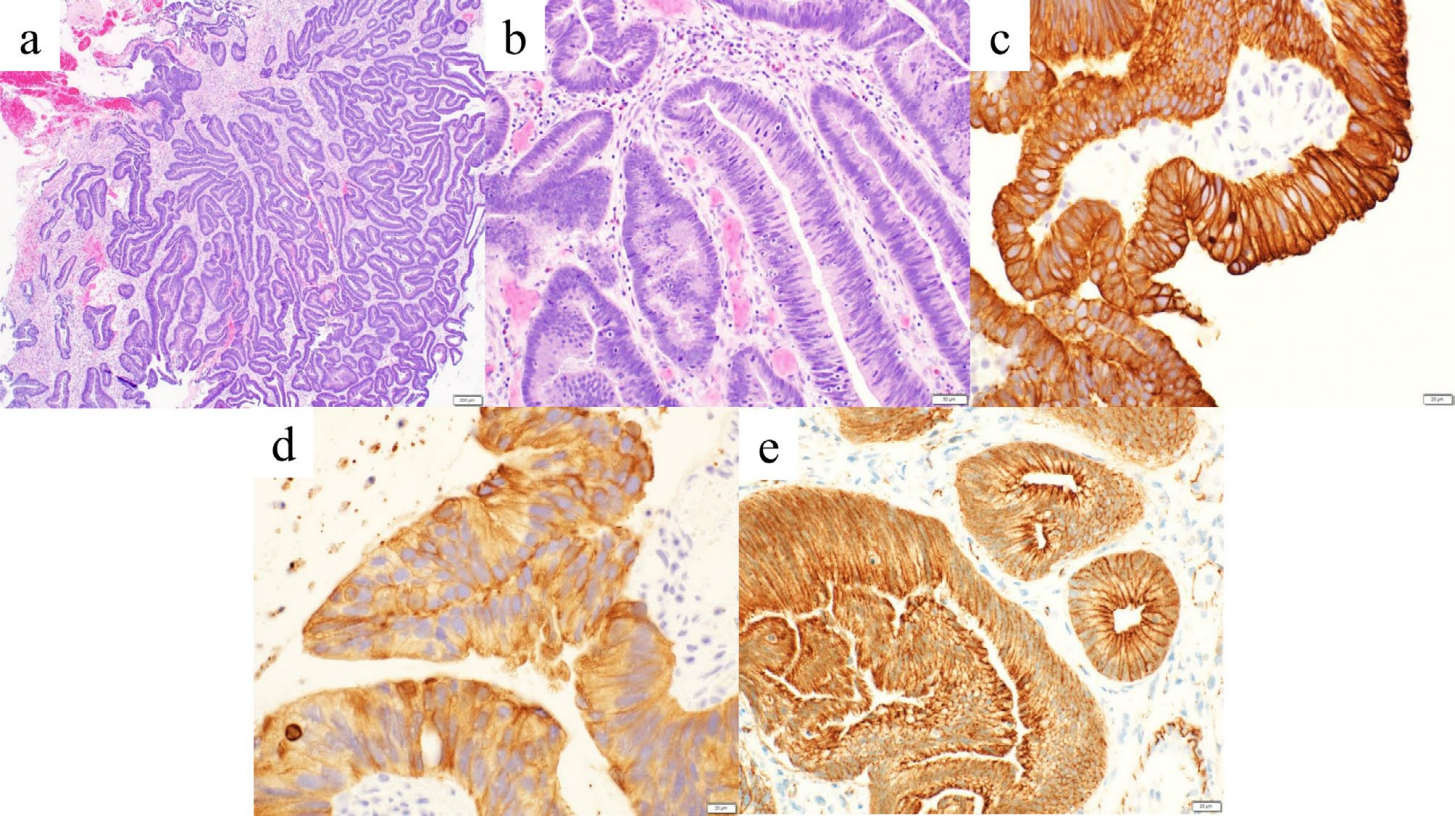
Histopathological and immunohistochemical findings of the tumor. (a) Hematoxylin and eosin staining (low magnification) showing infiltrating mucin‐producing adenocarcinoma arising from the bladder dome. Abundant extracellular mucin pools and gland‐forming tumor cells are observed. (b) High‐magnification view demonstrating columnar atypical epithelium with cytoplasmic mucin and focal signet‐ring–like morphology. (c) Immunohistochemical staining for CK7 showing diffuse cytoplasmic positivity in tumor cells, supporting a urachal/bladder‐type adenocarcinoma phenotype. (d) CK20 immunostaining demonstrating strong cytoplasmic positivity in the neoplastic glands. (e) β‐catenin immunohistochemistry revealing membranous staining without nuclear accumulation, effectively excluding colorectal adenocarcinoma metastasis. Overall, the histologic and immunophenotypic features are consistent with primary urachal adenocarcinoma.

Histopathology demonstrated invasive adenocarcinoma with mucin pools and focal signet‐ring features. Immunohistochemistry showed CK7 and CK20 positivity and membranous—without nuclear—β‐catenin expression, confirming a urachal/bladder‐type adenocarcinoma phenotype (Figure 2a–e).

**FIGURE 3:**
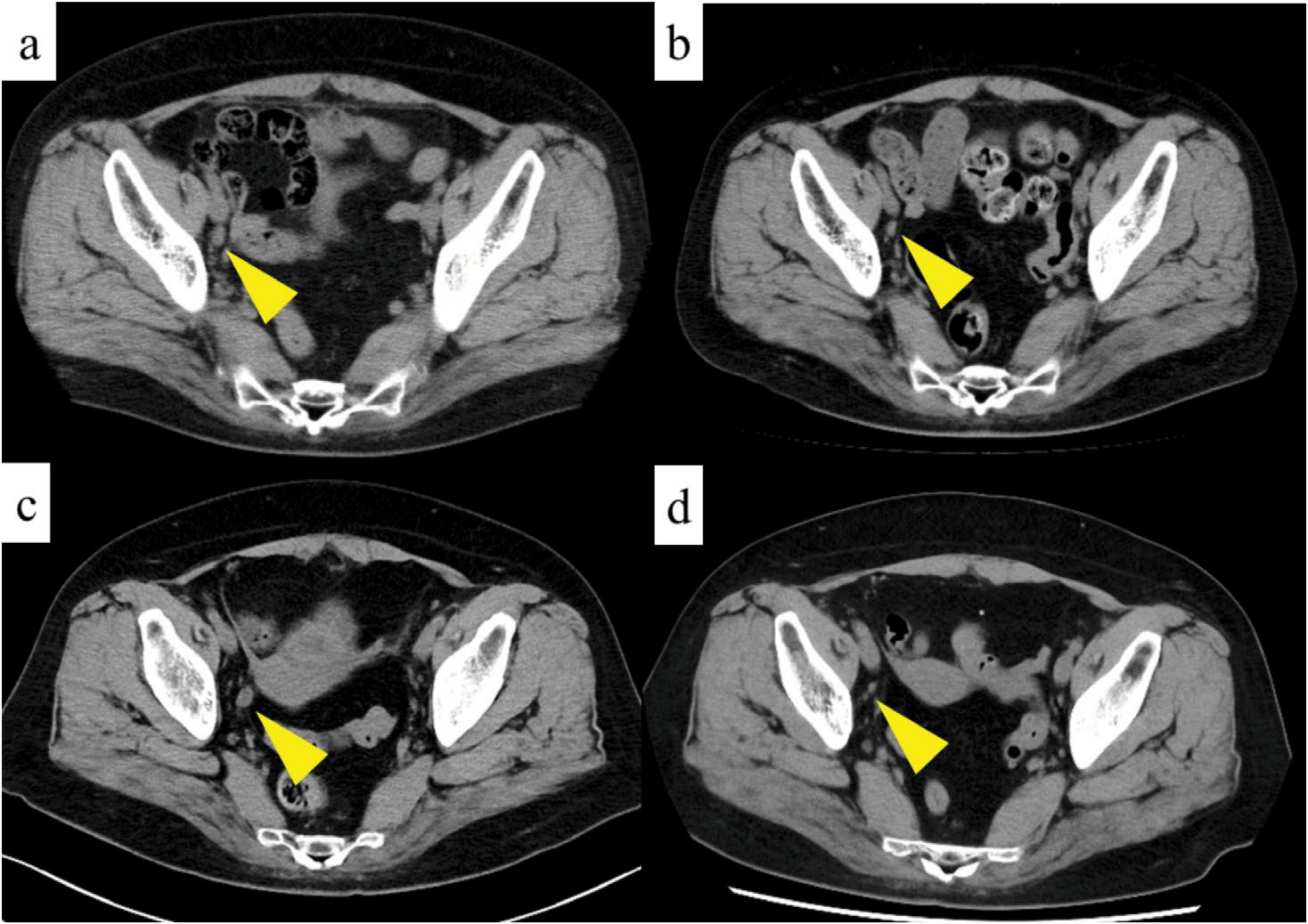
Sequential radiologic changes of the right obturator lymph node metastasis. (a) Baseline axial CT showing an enlarged right obturator lymph node. (b) After four cycles of gemcitabine–cisplatin, the right obturator lymph node showed slight interval reduction; however, the overall radiologic response remained stable disease. (c) After seven cycles of gemcitabine–cisplatin, the lesion enlarged compared with the post‐treatment nadir, fulfilling radiologic criteria for progressive disease. (d) Following pembrolizumab initiation, the right obturator lymph node decreased from 20 mm in short‐axis diameter at treatment initiation to 5 mm at 12 months, corresponding to a 75% reduction and a partial response according to RECIST 1.1.

Computed tomography (CT) revealed a bladder dome mass with peritoneal dissemination and an enlarged right obturator lymph node, corresponding to stage T4N1M0 disease. The baseline CT also showed a measurable obturator node (Figure 3a).

Gemcitabine–cisplatin (GC) therapy was initiated, achieving radiologic stable disease for four cycles; CT after four cycles showed slight reduction of the obturator node without meeting criteria for partial response (Figure 3b). After seven cycles at 8 months, follow‐up CT demonstrated enlargement of the obturator lymph node compared with baseline, meeting radiologic criteria for progressive disease (Figure 3c).

The primary dome lesion was not reliably assessable on non‐contrast follow‐up CT due to indistinct margins and mucinous composition and was considered non‐measurable.

Pembrolizumab (200 mg every three weeks) was started in July 2022. Because pembrolizumab was not approved for urachal carcinoma in Japan, it was administered off‐label after approval through the institutional Pharmaceutical Affairs Committee and after obtaining informed consent from the patient. At the initiation of pembrolizumab, the target right obturator lymph node measured 20 mm in short‐axis diameter. Serial CT showed gradual shrinkage, and the lymph node decreased to 5 mm at month 12, corresponding to a 75% reduction and a partial response according to RECIST 1.1. No immune‐related adverse events were observed (Figure 3d).

**FIGURE 4:**
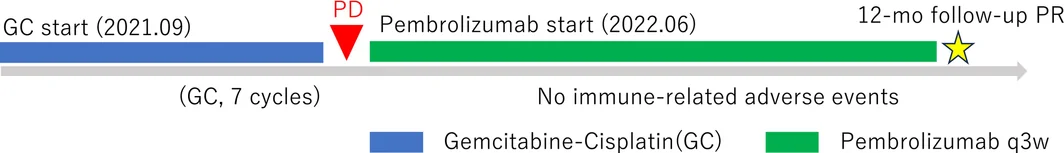
Clinical course timeline of systemic treatment. The timeline illustrates the therapeutic sequence and response. The patient received gemcitabine–cisplatin (GC) combination therapy for seven cycles during 2021, followed by radiologically confirmed progressive disease (PD). Pembrolizumab (200 mg every 3 weeks) was initiated in mid‐2021, leading to gradual tumor shrinkage and a durable partial response maintained for over 12 months without immune‐related adverse events.

Molecular profiling demonstrated microsatellite stability, low tumor mutational burden, and proficient mismatch repair; PD‐L1 testing was not possible due to limited tissue. The patient remained progression‐free for over 12 months until transfer to another facility (Figure 4).

## 3 Discussion

Owing to the rarity of UrC, no standard systemic therapy exists, and management is largely extrapolated from urothelial carcinoma [1, 4]. GC is commonly used as first‐line therapy [4], but responses are short‐lived, with median progression‐free survival of 6–10 months [5, 7]. Progression after seven GC cycles in our patient was consistent with previous reports [2, 13].

The role of ICIs in UrC remains unclear. Limited reports describe durable pembrolizumab or nivolumab responses, mostly in tumors with high PD‐L1 expression, mismatch repair deficiency, or high TMB [10, 11, 12]. For example, Chen et al. reported a high–PD‐L1 tumor with a sustained response [11]. In contrast, our patient achieved a durable partial response for over 12 months despite microsatellite stability and low TMB; PD‐L1 status could not be assessed because of insufficient tissue. This suggests that ICI benefit in UrC may not be restricted to tumors with MSI‐H or high TMB [12].

Several mechanisms may explain this response. Molecular profiling shows UrC frequently harbors KRAS, GNAS, and TP53 mutations but generally has lower TMB than urothelial carcinoma [9]. Even low‐TMB tumors can express neoantigens capable of activating T cells. Prior cytotoxic chemotherapy may also have promoted an immune‐permissive microenvironment; platinum agents induce immunogenic cell death and enhance antigen presentation, potentially priming tumors for immunotherapy [14]. PD‐L1 evaluation was not possible in this case, but prior studies show heterogeneous PD‐L1 expression without consistent correlation to response [4]. Combined with the microsatellite‐stable, low‐TMB profile here, this suggests that durable responses to PD‐1 blockade can occur even without conventional biomarkers, consistent with reports of microsatellite‐stable, low‐TMB tumors benefiting from ICIs through PD‐L1–independent mechanisms [15].

Clinically, this case offers several insights. First, this case, together with previous reports, suggests that pembrolizumab may provide durable clinical benefit in selected patients with platinum‐refractory UrC [10, 11]. However, immunotherapy has not been established as a standard treatment for platinum‐refractory UrC. The absence of immune‐related toxicity supports the safety and feasibility of pembrolizumab in this setting. Second, microsatellite stability or low TMB alone may not preclude a response to ICI therapy, although predictive biomarkers for ICI efficacy in UrC remain to be established. Third, longitudinal imaging is important during ICI therapy, as delayed or gradual responses may appear after months of apparent stability. Such late‐onset responses are recognized in other malignancies but remain rarely reported in UrC [11, 12].

Integrating genomic and immunologic profiling will be crucial to refine patient selection. Urachal carcinoma shares molecular features with colorectal and urothelial adenocarcinomas, such as MAPK and Wnt pathway activation [4, 9]. Preclinical and clinical data from other cancers suggest that combining ICIs with targeted or cytotoxic agents may further enhance antitumor immunity [16]. Future studies evaluating the tumor microenvironment—particularly T‐cell infiltration, PD‐L2 expression, and tertiary lymphoid structures—may help identify tumors that remain immunologically active despite the absence of currently established favorable biomarkers. Establishing multicenter registries for urachal and other rare genitourinary adenocarcinomas will also be critical for generating meaningful datasets and advancing biomarker‐driven therapeutic development [16].

A limitation of this report is the lack of PD‐L1, PD‐L2, and immune cell profiling, which restricts mechanistic interpretation. However, the durable response in a microsatellite‐stable, low‐TMB tumor suggests potential immune responsiveness beyond conventional biomarkers.

Overall, this case illustrates that the therapeutic benefits of immune checkpoint inhibitors may extend beyond established biomarker boundaries. Given the rarity of urachal carcinoma, accumulating well‐characterized long‐term responders—including biomarker‐negative cases—will be essential for refining predictive models and guiding precision immunotherapy in this rare non‐urothelial malignancy.

## 4 Conclusion

Pembrolizumab achieved durable disease control in metastatic urachal carcinoma refractory to platinum chemotherapy, despite microsatellite stability and low tumor mutational burden. This case suggests that PD‐1 blockade may have clinical activity in selected patients with urachal carcinoma despite microsatellite stability and low tumor mutational burden.

## Ethics Statement

According to the policy of our institution, formal IRB review is not required for single‐patient case reports that do not disclose identifiable private information.

## Consent

Written informed consent for publication, including images, was obtained from the patient and is available to the Editorial Office upon request.

## Conflicts of Interest

The authors declare no conflicts of interest.

## Data Availability

The authors have nothing to report.
